# Brain Targeting of Quetiapine Fumarate via Intranasal Delivery of Loaded Lipospheres: Fabrication, In-Vitro Evaluation, Optimization, and In-Vivo Assessment

**DOI:** 10.3390/ph15091083

**Published:** 2022-08-30

**Authors:** Randa Mohammed Zaki, Mohammed F. Aldawsari, Manal A. Alossaimi, Shaikah F. Alzaid, Vidya Devanathadesikan Seshadri, Alanood S. Almurshedi, Basmah Nasser Aldosari, Rehab Mohammad Yusif, Ossama M. Sayed

**Affiliations:** 1 Department of Pharmaceutics, College of Pharmacy, Prince Sattam Bin Abdulaziz University, P.O. Box 173, Al-Kharj 11942, Saudi Arabia; 2 Department of Pharmaceutics and Industrial Pharmacy, Faculty of Pharmacy, Beni-Suef University, Beni-Suef P.O. Box 62514, Egypt; 3 Department of Pharmaceutical Chemistry, College of Pharmacy, Prince Sattam Bin Abdulaziz University, P.O. Box 173, Al-Kharj 11942, Saudi Arabia; 4 Department of Pharmacology and Toxicology, College of Pharmacy, Prince Sattam Bin Abdulaziz University, P.O. Box 173, Al-Kharj 11942, Saudi Arabia; 5 Department of Pharmaceutics, College of Pharmacy, King Saud University, P.O. Box 2457, Riyadh 11451, Saudi Arabia; 6 Department of Pharmaceutics, Faculty of Pharmacy, Mansoura University, Mansoura 35516, Egypt; 7 Department of Pharmaceutics and Pharmaceutical Technology, College of Pharmacy, Taibah University, Al-Madinah Al-Munawarah 41411, Saudi Arabia; 8 Department of Pharmaceutics, Faculty of Pharmacy, Sinai University-Kantara Branch, Ismailia 41612, Egypt

**Keywords:** intranasal, brain targeting, lipospheres, quetiapine fumarate, DTE%

## Abstract

A liposphere system for intranasal delivery of quetiapine fumarate (QTF) was created to assess the potential for enhanced drug delivery. We investigated the effects of particle size, entrapment effectiveness, poly dispersibility index, and pluronic incorporation percentage on these variables. The optimal formula was examined using a TEM, and investigations into DSC, XRD, and FTIR were made. Optimized liposphere formulation in vitro dissolution investigation with a mean diameter of 294.4 ± 18.2 nm revealed about 80% drug release in 6 h. The intranasal injection of QTF-loaded lipospheres showed a shorter T_max_ compared to that of intranasal and oral suspension, per the findings of an in vivo tissue distribution investigation in Wistar mice. Lipospheres were able to achieve higher drug transport efficiency (DTE %) and direct nose-to-brain drug transfer (DTP %). A potentially effective method for delivering QTF to specific brain regions is the liposphere system.

## 1. Introduction

One percent of the world′s population suffers from schizophrenia, a chronic psychotic disorder. Symptoms of schizophrenia typically develop in adulthood and persist for the rest of a person’s life. Antipsychotic medications, especially atypical antipsychotic medications, can effectively diminish both the positive (delusions, auditory illusions) and adverse (social disengagement, grossly disorganized, inability to pay attention) symptoms of schizophrenia [1].

Quetiapine (QTF) is an atypical antipsychotic medication that is thought to have a broader efficacy than standard antipsychotics and many other atypical antipsychotic medicines [2]. 2-[2-(4-dibenzo[b,f][1,4]thiazepin-11-yl-1-piperazinyl)ethoxy]-ethanol is a dibenzothiazepine derivative with the chemical name 2-[2-(4-dibenzo [b,f][1,4]thiazepin-11-yl-1-piperazinyl)ethoxy]-ethanol.

QTF’s exact mode of action is uncertain, although it is thought to block neuron receptors for multiple neurotransmitters, preventing nerves from communicating with one another. The action is assumed to be mediated by antagonistic interactions between dopamine type 2 and serotonin type 2 (5HT2) receptors. QTF has antidepressant properties, which are likely to be mediated in part by its metabolite N-des alkyl quetiapine fumarate, which inhibits selective norepinephrine reuptake and activates the 5-HT1A and 5-HT7 receptors [3]. QTF also has a favorable safety record, as evidenced by numerous past clinical trials [4]. Many people with schizophrenia have stated that QTF enhances their cognitive abilities, and it is particularly well-tolerated in the elderly [5]. QTF has been licensed as a first-line treatment for schizophrenia due to its efficacy against a variety of illnesses [6,7]. QTF is also said to be effective and tolerable in the treatment of bipolar mania [8,9]. However, certain QTF constraints make it difficult to provide it via the traditional manner. QTF is a lipophilic medication with a low water solubility and a low bioavailability (5–15%) after oral administration [10]. The liver metabolizes it significantly [11,12,13]. As a result, a technique can be devised to increase QTF bioavailability while bypassing first-pass metabolism. Because QTF’s target site is the brain, a delivery system that delivers QTF directly to the brain can be devised.

Effective brain targeting can lead to increased drug concentrations in the brain, avoiding first-pass metabolism and lowering therapeutic doses. It was thought that developing a brain targeting formulation of QTF would be particularly beneficial in the clinical therapy of schizophrenia.

The technique of getting drugs into brain tissue is challenging. The blood–brain barrier prevents the majority of chemicals from effectively reaching the brain. Although intracerebroventricular or intraparenchymal injections can deliver medications directly to the brain, doing so repeatedly is risky, costly, and necessitates surgical intervention [14]. The advantages of intranasal administration can outweigh the disadvantages of other administration methods and allow for tailored delivery to the brain [15]. Intranasal administration has been shown in previous research [16,17] to be a practical, uncomplicated, non-invasive, and comfortable alternative route of administration, with quick drug transport to the brain and improved therapeutic efficacy [17,18,19] of the drug. Following intranasal administration, it is seen that the drug is delivered to the brain in greater quantity and more swiftly [18]. The intranasal method of drug delivery to the brain is more promising than the intravenous and oral routes of administration [20,21]. The formulation can be developed to target QTF through the olfactory part of the nasal cavity, allowing it to reach the brain quickly.

The olfactory epithelium, which serves as a doorway for chemicals entering the CNS and peripheral circulation, transports drugs from the nose to the brain. Both an intra-neuronal and extra neuronal channel into the brain is provided by the olfactory system [22,23]. Axonal transport is involved in the intraneuronal pathway, which takes hours to days for medications to reach different brain regions. The extra neuronal pathway, on the other hand, relies on bulk flow transfer through perineural channels, which deliver medicines directly to brain parenchymal tissues and/or CSF. As a result of the extra neuronal route, medicines can reach the CNS in minutes. The medicine must travel through the mucus to be absorbed through the nasal mucosa. Small uncharged particles easily pass through the mucosa, whereas large or charged particles have a tough time doing so. The drug may be absorbed from the mucosa by simple diffusion across the membrane, paracellular transport by movement between cells, or transcytosis by vesicle carriers after passing through mucus [23].

Although the intranasal route is effective for topical, systemic, and CNS medication delivery, it is ineffective for many others due to low nasal bioavailability. Low drug solubility, fast enzymatic breakdown in the nasal cavity, poor membrane penetration, and quick mucociliary clearance all limit the bioavailability of nasally given medicines [24].

Drug delivery systems with a size below 1000 nm are known as nanomedicine formulations. These formulations are made from a variety of raw materials, including phospholipids (liposomes), lipids (SLN, NLC), and polymers (nanocapsules, nanospheres, micelles). Although spherical shapes are preferred for some purposes, they could have other shapes, sizes, and surface properties. In pre-clinical research, many nanosystems were examined, from in vitro, ex vivo, and in vivo tests on the health or pathological model of animals to screening of a suitable formulation to reach the brain intra-nasally [25].

To load neuroactive medications such as dimethylfumarate, retinyl palmitate, progesterone, and the endocannabinoid hydrolysis inhibitor URB597, lipid NPs were made using tristearin in combination with gliceryl monoolein [26]. Three distinct forms of SLN were loaded with dimethyl fumarate as a possible multiple sclerosis treatment. After intraperitoneal or IN injection, Esposito et al. investigated the biodistribution of polysorbate 80-treated SLN using fluorescence imaging in mice [26]. Shah and colleagues investigated rivastigmine-loaded SLN that was DoE-optimized utilizing several lipids (Apifil, Compritol glycerylmonostearate, stearic acid). The authors of this work did a histopathological analysis on the nasal mucosa in preparation for possible IN usage [27].

Nanomedicine and IN administration were researched to achieve two objectives: quick delivery to the brain for acute treatment or sustained drug release and fewer administrations for chronic treatment. Clonazepam microemulsions were developed by Vyes Tushar et al. for IN administration to the brain. A comparison of the drug’s biodistribution in the brain following IN and IV doses was performed on Swiss albino rats as part of their study of the drug’s radiolabeled analogue using 99mTc (technetium). Their findings demonstrated that brain/blood uptake ratios indicated more effective targeting with IN administration and optimum brain targeting with an IN clonazepam loaded microemulsion at 30 min after IN or IV administrations. In comparison to IV administration, the brain/blood ratio was greater at all sampling points after 8 h from IN treatment, indicating that the molecules were distributed widely throughout the brain [18].

Lipospheres, similar to solid lipid nanoparticles, are one of the preferred carriers for topically delivered medications since their lipid components have an approved status or are excipients utilized in commercially available topical cosmetic or pharmaceutical formulations. The stratum corneum can be directly contacted by the small lipid particles, increasing the amount of medication that gets to the mucosa or skin. Due to the solid lipid matrix of these carriers, controlled release is possible, which is essential for providing the drug over an extended period of time, lowering systemic absorption, and enhancing drug stability [28].

The purpose of this study was to develop QTF lipospheres for intranasal delivery of QTF to the brain. By avoiding first-pass metabolism, these lipospheres can carry QTF to the brain more quickly and effectively while also reducing the drug’s dose and side effects.

## 2. Results and Discussion

An exceptional lipid-based carrier system is demonstrated by lipospheres, which have a phospholipid coat embedded in their surface and a lipid core stabilized by it. It has been found that several formulation factors have an impact on the drug’s size, release profile, and entrapment inside the lipospheres.

### 2.1. Trial Formulations

Table 1 shows the EE% data for the trial formulation of different lipospheres. It was obvious that increasing the drug: lipid ratio from 1:2 to 1:10 leads to an increase in EE% to 1:8 ratio; then, there was a decrease in the EE at 1:10 ratio. The initial increase in EE% could be attributed to the increase of the inter lipid layers spaces available to accommodate drug molecules [29,30]. A further increase in lipid content led to a decrease in EE, which could be attributed to the increase of viscosity during emulsification and increased porosity of the particles, and which lead to more drug escaping the encapsulation [31].

The increase of lipid ratio from 1:2 to 1:10 led to an obvious increase in particle size, which was in accordance with the previous literature [29]. The increase in particles size was parallel to an increase in PDI, which was attributed to the increased viscosity of the emulsion which led to increased conjugation of particles.

Zeta potential of formed particles showed a high negative charge due to the presence of large proportions of negatively charged excipients used in the formulations. The negative charge originated from the use of phosphatidyl choline and stearic acid. In addition, using pluronic led to increase of the negative charge. This high negative charge is preferable for the stability of the formed suspension.

### 2.2. Formulation’s Design

A drug lipid ratio of 1:8 was chosen for the design because this ratio achieved the highest EE.

#### 2.2.1. Entrapment Efficiency

**Figure 1:**
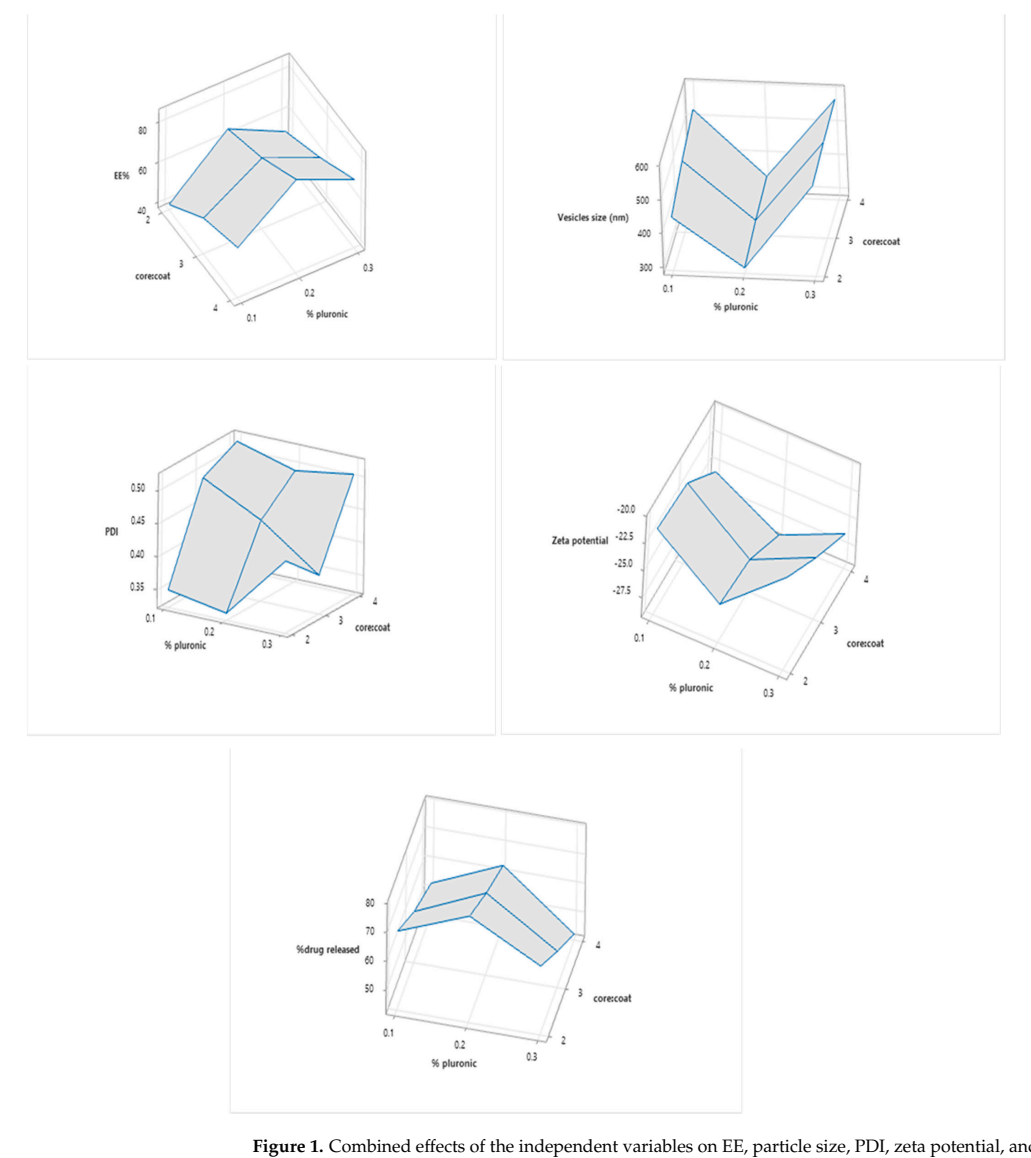
Combined effects of the independent variables on EE, particle size, PDI, zeta potential, and % drug released.

EE data of formulated lipospheres are shown in Table 2. The combined effects of the independent variables on EE are illustrated in Figure 1. Increasing the core:coat ratio led to an increase in the EE of lipospheres, which could be attributed to the increased the amount of stearic acid proportions, which could accommodate more drugs [32,33]. Increasing the concentration of pluronic led to an increase in EE moving from 0.1 to 0.2%, followed by a decrease in EE at 0.3% (83.841 ± 1.086 vs. 71.409 ± 0.294%). This decrease may be linked to the increase of particle size [32,34].

#### 2.2.2. Particle Size

Particle size data of formulated lipospheres are shown in Table 2. The combined effects of the independent variables on particle size are illustrated in Figure 1. Increasing the core:coat ratio from 2:1 to 4:1 led to a significant increase in particle size of liposphere in each level of pluronic concentration. This was in accordance with the previous literature where increasing core stearic acid proportions led to an increase in emulsion viscosity and larger droplets [34,35].

On the other hand, increasing pluronic concentration lead to a decrease in particle size of lipospheres due to increasing the emulsification efficiency. However, increasing the pluronic level to 0.3% led to slight increase in the particle size. This increase could be attributed to the increased viscosity of the system.

The polydispersity index (PDI) of the formulated lipospheres was linked to both particle size and zeta potential. It was significantly small (*p* < 0.05) at 0.333 ± 0.1697 in the case of formula F4, with the smallest particle size.

#### 2.2.3. Zeta Potential

From data shown in Table 2 and Figure 1, all lipospheres carried a negative charge ranging from −21.35 ± 2.8991 to −28.8 ± 1.9798 mV. There was no significant difference (*p* > 0.05) between the different formulations in terms of zeta potential. The negative surface charge could be attributed to the high charges of stearic acid and lecithin. High zeta negative zeta potential ensures good repulsion forces between lipospheres and optimum physical stability of the formulations.

#### 2.2.4. Drug Release Profiles and Kinetics

**Figure 2:**
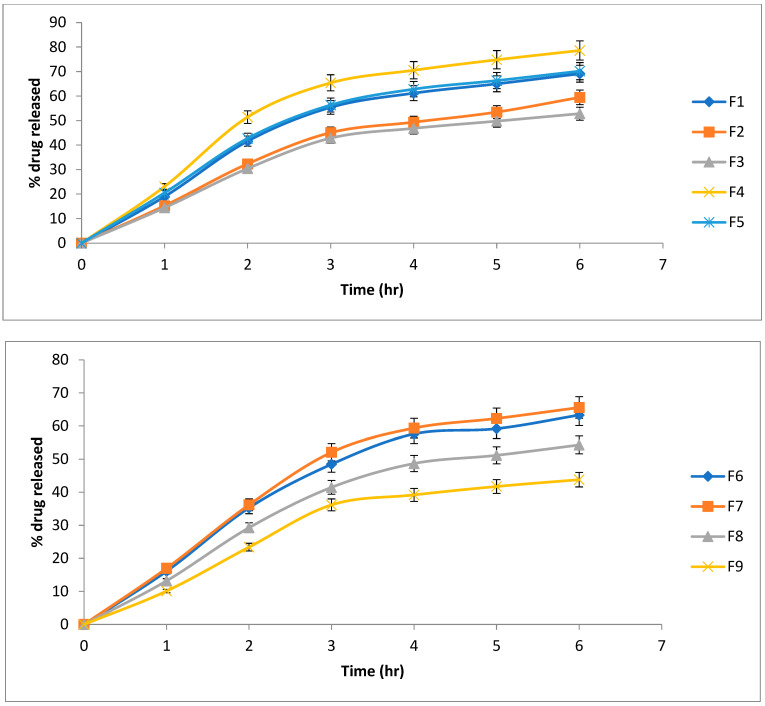
Release profiles of QT from formulated lipospheres (mean ± SD).

QTF Release profiles are illustrated in Figure 2. Cumulative present drugs released are shown in Table 2. Release kinetics of QTF from different lipospheres are shown in Table 3. All release profiles showed a steep increase of QTF release from lipospheres, which slowed down at the end of the 6 h period of the study. There is an inverse proportion relationship between the core:coat ratio and the cumulative QTF released due to the increased packing of the lipid content [32,34]. All formulations showed a release obeying Higuchi diffusion model. High values of pluronic lead to decreased release percentage due to the increase in particle size.

Formula F4 showed the highest release of QT over the 6 h period (78.60%) compared to other formulations (*p* > 0.05), and was chosen for further investigation.

### 2.3. Powder X-ray Diffraction Examination

**Figure 3:**
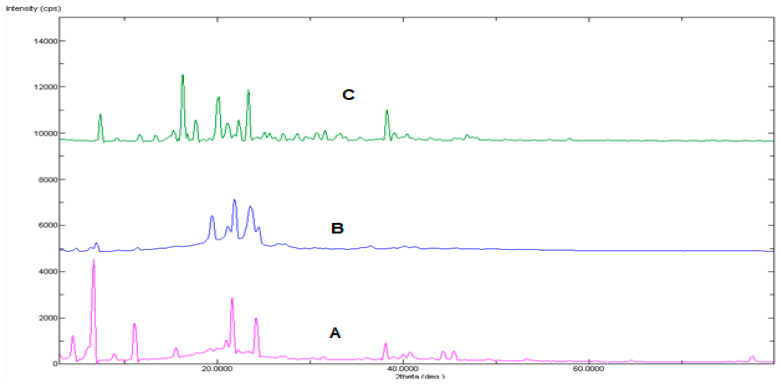
XRD of A: pure QTF, B: Lecithin, stearic acid, and Pluronic F-127 mixture, and C: the best formula.

Powder X-ray diffraction graphs of QTF, lLecithin, stearic acid, and pluronic F-127 mixture and formula F4 are shown in Figure 3. The characteristic peaks of QTF were significantly reduced and shallowed by being incorporated into lipospheres. Loss of drug crystallinity offers a potential advantage regarding release of the drug from liposphere carriers into cellular domains [36,37].

### 2.4. Differential Scanning Calorimetry

**Figure 4:**
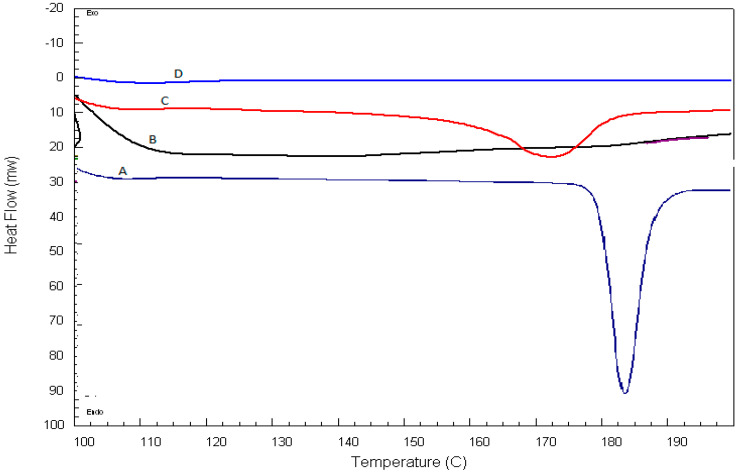
DSC thermograms of A: Pure QTF, B: Lecithin, stearic acid, and Pluronic F-127 mixture, C: Lecithin, stearic acid, Pluronic F-127, and QTF, and D: the best formula.

DSC thermograms of QTF, excipient mixture, and QTF excipient physical mixture are illustrated in Figure 4. QTF showed a strong endothermic peak at 183 °C, which reflects strong drug crystallinity. Incorporating QTF into lipospheres led to the disappearance of the drug peaks, which can be attributed to the transition of the crystalline state into an amorphous one or the dilution of the drug with the excipients [36].

### 2.5. Transmission Electron Microscopy

**Figure 5:**
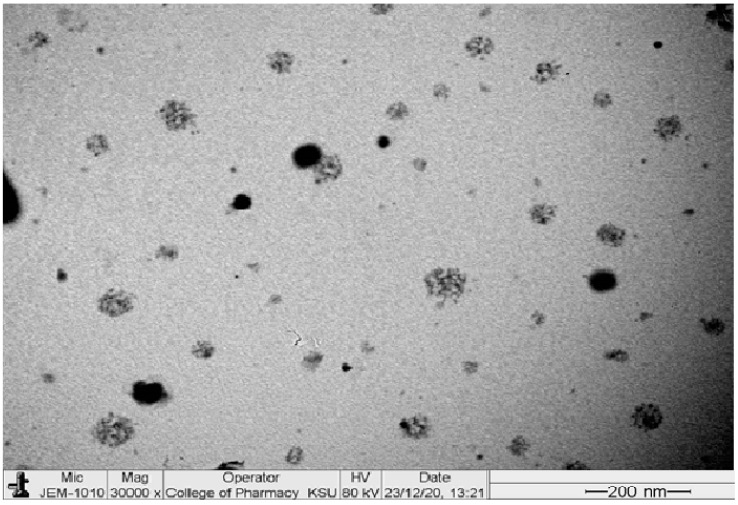
TEM image of the optimum formula.

TEM images of formula F4 is shown in Figure 5. The particles have an uneven surface and are typically spherical in shape when phosphatidylcholine is utilized as the coating [38,39,40]. Additionally, lipospheres showed an average dimension of 278.92 ± 24.11 nm, which is near to size data obtained from the DLS.

### 2.6. Stability Study

As shown in Table 4, there were no substantial changes in particle size, zeta potential, and EE% at both 7 and 30 days. This reveals the good stability of the optimum formula through storage for one month at 4 °C.

### 2.7. In Vivo Study

**Figure 6:**
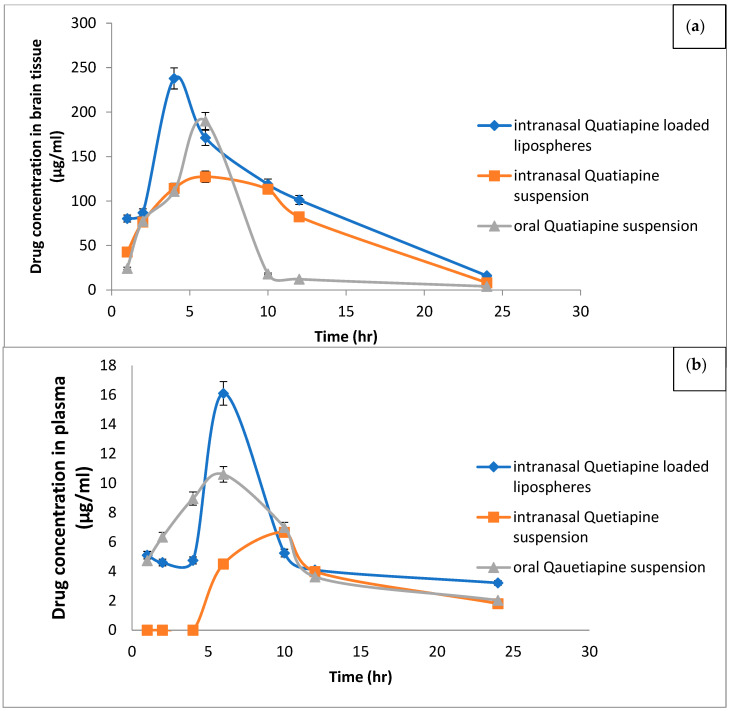
QTF concentration levels in (**a**) the brain and (**b**) plasma in rats.

Quantitative analysis of QTF in plasma and the brain were performed to ascertain the advantages of drug encapsulation in the lipospheres system. Figure 6 compares intranasal QTF suspension and oral QTF suspension to the mean QTF concentration in plasma and brain concentration-time of rats after intranasal administration of the optimal liposphere formula. The corresponding calculated pharmacokinetic and brain target parameters of QTF (i.e: C_max_, T_max_, t1/2, AUC, F %, DPT % and DET %) are listed in Table 5. The highest concentration in plasma (C_max_) (22.08 ± 10.23 µg/mL), (6.67 ± 1.37 µg/mL) and (10.87 ± 0.93 µg/mL) was reached within six, ten, and seven hours following administration of I.N lipospheres, I.N, QTF suspension, and Oral QTF suspension, respectively. The AUC_0-24_ h and relative bioavailability of QTF in rat plasma from IN Liposphere suspension and I.N. QTF suspension were 133.65 ± 16.5 µg h/gm and 109.05%, 79.09 ± 12.52 µg h/gm and 65.14%, respectively, compared with oral QTF suspension.

Figure 5 compares the mean QTF concentration in the brain of rats given the oral QTF suspension with the I.N. QTF suspension and I.N. QTF liposphere suspension formulation. Data revealed that the I.N. QTF liposphere formulation exhibited significantly higher values for C_max_ and AUC_0-24_hr (237.86 ± 34.01 µg/mL and 2361.04 ± 279.46 µg h/gm) compared with I.N. QTF suspension (160 ± 13.67 ng/g and 36,850 ± 200.36 µg h/gm) with relative bioavailability equal to 215.97% and 154.96%, respectively, compared with oral QTF suspension (*p* < 0.05). Additionally, the MRT values in rat brain for IN QTF lipospheres and IN QTF suspension were significantly higher than that for oral QTF suspension with values 9.41 ± 0.47, 9.14 ± 0.34 and 7.42 ± 0.50 h, respectively (*p* < 0.05). The intranasal administration of IN QTF lipospheres exhibited nearly 1.69, 1.39- and 1.02-fold increase in the C_max_, AUC0–∞ and MRT, respectively, compared to intranasal QTF suspension; hence the IN QTF lipospheres were superior to I.N. drug suspension in targeting QTF to the brain (*p* < 0.05) [24,41].

Brain targeting efficiency was estimated according to of % drug targeting efficiency (%DTE) and drug transport % (DTP). The higher % DTE (228.36, 169.66) and positive % DTP (51.72, 48.82) were observed for IN QTF lipospheres and IN QTF suspension, respectively. The % DTE findings, which were greater than 100, guaranteed that IN QTF lipospheres would carry more medication to the brain than oral dosing. The preferred method of brain drug delivery can be related to the route of administration and the form of the drug, where the formula delivered via the intranasal route in the form of a nano colloidal lipophilic carrier can be delivered directly to the brain by using direct routes of the olfactory and trigeminal nerves without crossing the BBB, whereas the QTF suspension administered orally or intravenously has no direct pathway to the brain and must cross the BBB.

To investigate the pathway of QTF uptake into the brain from the nasal mucosa, the % DTP of IN QTF Lipospheres and IN QTF suspension was calculated. Positive values of the % DTE (51.72, 48.82) of IN QTF lipospheres and IN QTF suspension indicate a direct QTF uptake from the nasal cavity to the CSF and/or brain tissue via the trigeminal nerve and olfactory, which reach from the nasal cavity into the brain away from the BBB (direct nose-to-brain routes). The results agree with the previously reported works by Abdel, Bary et al., 2013 and Pailla et al., 2019 [42,43].

The enhanced delivery of QTF from lipospheres to the brain followed by intranasal administration of QTF lipospheres is attributed to: (1) Due to their high lipid content, lipospheres are known to exhibit superior BBB penetration when compared to free drug forms. This can facilitate greater transcellular diffusion over the BBB. The surfactants utilized in this work, particularly P glycoprotein, can function as uptake enhancers, decrease nanoparticle clearance by the reticuloendothelial system, and diminish the efflux system. (2) The direct passage of the lipospheres from the nose to the brain is responsible for their increased brain delivery; and (3) the increased concentration gradient from the systemic circulation to the brain can provide a greater gradient for the diffusion across the BBB due to the increased absolute bioavailability and higher QTF plasma concentrations.

## 3. Material and Methods

### 3.1. Material

Quetiapine fumarate was a gift from the Al-Jazera company for pharmaceuticals. L-α-Phosphatidylcholine, type X-E: from dried egg yolk, stearic acid, and stearyl alcohol was purchased from Sigma Chemical Co., St. Louis, MO, USA. Tristearin was purchased from Fluka Chemical Co., Buchs, Switzerland. Soybean lecithin (Phospholipon^®^ 90G) was purchased from Nattermann, Cologne, Germany (PC content 94–102%). Chloroform, absolute ethyl alcohol, potassium dihydrogen phosphate, disodium hydrogen phosphate, and sodium chloride were purchased from Adwic, El-Nasr chemical Co., Cairo, Egypt, according to the methods of Prolabo, Paris, France. Spectra/Por dialysis membrane, 12.000–14.000 molecular weight cut off was purchased from Spectrum Laboratories Inc., Rancho Dominguez, Canada.

### 3.2. Animals

Male Wister albino rats (140 ± 20 g) procured from the Animal house of Prince Sattam bin Abdul Aziz University were used for the study. The animals were housed in large polypropylene cages in a temperature-controlled room (22 ± 2 °C) and provided with standardized pellet feed and clean drinking water ad libitum. The study received clearance from the Institutional Animal Ethical Committee (IAEC) number 202010001 of CPCSEA (Committee for the Purpose of Control and Supervision of Experiments on Animals).

### 3.3. Study Design

Five trial formulations were prepared based on studying the effect of changing the ratio of drug:lipid at five levels (1:2, 1:4, 1:6, 1:8, and 1:10). Based on the results obtained from the trial formulations, full 3^2^ factorial design was constructed using two independent variables (Core:Coat and Pluronic percentage *w/w*) at three levels.

### 3.4. Preparation of QT Loaded Lipospheres

QTF-loaded lipospheres were prepared by a high-speed homogenization method. Firstly, an accurate weight of QTF (100 mg) and lecithin were dissolved in 5 mL methanol. Then, stearic acid was dissolved in 5 mL acetone. The methanolic solution was mixed with acetone solution, then this mixture was added dropwise to 10 mL preheated Pluronic F127 solution at 700 C followed by homogenization at 9000 rpm for 10 min at the same temperature. Finally, it was sonicated for 15 min to inhibit lipid crystallization then allowed to be cooled to room temperature with continuous stirring for 2 h.

### 3.5. Separation of Unentrapped QTF from the Prepared Lipospheres

QTF lipospheres were separated from free unentrapped QTF by centrifugation at 20,000 rpm for 30 min at 4 °C [38,44,45,46]. The pellets formed were washed with 10 mL phosphate buffered saline and recentrifuged again for 30 min. The washing of pellets was repeated in triplicate to ensure the complete removal of the un-entrapped drug. The lipospheres were decanted and kept in the refrigerator for further investigations.

### 3.6. Determination of Entrapment Efficiency.

The concentration of the entrapped drug was measured by sonication and lysis of the lipospheres with 100% alcohol [38]. To prevent evaporation, a precisely weighed amount of loaded lipospheres (50 mg) was dissolved in 10 mL pure alcohol and covered well with parafilm.

To get a clear solution, the solution was sonicated for 15 min. In total, 1 mL of this solution was added to 9 mL of pure alcohol as an aliquot. For another 15 min, the solution was sonicated. After adequate dilution, the concentration of QTF in 100% alcohol was evaluated spectrophotometrically (Shimadzu, model UV-1601 PC, Kyoto, Japan) at 288 nm. At the same wavelength, unloaded lipospheres yielded minimal absorbance values. Each sample was examined three times.

The following relationship was used to calculate the entrapment efficiency:

(1) $$\text{Entrapment Efficiency Percentage}=\frac{Entrappted\;Drug\;}{Total\;Drug}\times 100$$

### 3.7. Characterization of QTF Lipospheres

#### 3.7.1. Morphological Description

TEM (TEM-1010, Tokyo, Japan) was used to describe the morphology as well as the dimensions of lipospheres. It was dropped onto the surface of a copper grid covered with carbon after sample preparation. To allow lipospheres to cling to carbon substrates, each sample was allowed to dry. We used a drop of 1% aqueous phosphotungestic acid dye to stain the grid, which was then air-dried for 2 min after excess dye was removed with filter paper. The stained sample was then examined and visualized using the TEM. The measurement was repeated six times to compute the average of lipospheres dimensions.

#### 3.7.2. Particle Size Analysis

The size of the lipospheres was evaluated by light scattering based on laser diffraction using the Malvern Master Sizer (Malvern Instruments Ltd., Worcestershire, UK) [11] laser diffraction particle size analyzer, and the distribution modal size was computed. Measurements were taken with a 45 mm focal objective, a 2.4 mm beam length, and obscuration levels ranging from 5 to 10%.

#### 3.7.3. Differential Scanning Calorimetry (DSC)

DSC analysis was performed on samples of QTF, plain, and drug-loaded lipospheres of the chosen formulation using a differential scanning calorimeter (Schimadzu, model TA-50 WSI, Kyoto, Japan) calibrated with indium. The test was done on 1 mg samples that were sealed in ordinary aluminium pans. Thermograms were produced using a dry nitrogen flow rate of (25 mL/min) and a scanning rate of 10 °C/min. Each sample was scanned at temperatures ranging from 0 to 200 °C.

#### 3.7.4. In Vitro Release of QTF from Lipospheres

The release of QTF from the produced lipospheres was evaluated using a molecular porous membrane (Spectra/Por dialysis membrane 12–14.000 M.wt cut off) and the membrane diffusion technique [6]. A precise amount of QTF lipospheres, equivalent to 2 mg QTF, was suspended in 1 mL phosphate buffered saline (pH 7.4) and transferred to a glass cylinder with a 7 cm length and 2.5 cm diameter. This cylinder was equipped with a presoaked dialysis membrane and suspended in a dissolution flask of a USP dissolution tester (Pharma Test, Hainburg, Germany) containing 100 mL phosphate buffered saline before adding the liposphere suspension (pH 7.4). The device was set to a constant speed of 50 revolutions per minute (rpm) and a temperature of 32 degrees Celsius. Over an 8 h period, samples were taken after 15 min, 30 min, 1 h, and every hour, and drug content was measured spectrophotometrically at 288 nm. The results were calculated as the average of three runs. To establish the order of release, the release data was submitted for kinetic treatment.

#### 3.7.5. Stability Study

The optimum QTF lipospheres were kept in an air-tight vial, kept away from light at 4 °C for one month [47]. Samples were withdrawn and evaluated for particles size, Zeta potential, and EE%.

### 3.8. In-Vivo Study

Wister albino mice were divided into seven groups of six animals each, for each formula of quetiapine. The first group served as normal control whereas the rest of the animals received an amount of formula equivalent to 20mg/kg. Animals in all the groups were fasted for 18 h prior to dosing with quetiapine formulae. Dosing of animals orally was done by the method of Kuentz, 2012 [48,49,50,51,52], and was followed for all the intranasal drug delivery of quetiapine. The dose of 20mg/kg was used for the study [51,53].

At different time intervals of 1, 2, 4, 6, 10,12, and 24 h following administration of oral QTF suspension, intranasal QTF suspension and intranasal QTF-loaded lipospheres, six animals were sacrificed from each group by cervical decapitation and blood was collected in commercially available anticoagulant-treated tubes for plasma separation.

#### 3.8.1. Separation of Plasma

Blood was collected in a commercially available anticoagulant-treated tube. The tubes containing spray-dried Heparin /EDTA anticoagulant is used in separation of plasma from blood. The tube was centrifuged at 2000× *g* for 10 min [54]. Cells are removed from plasma by centrifugation for 10 min at 1000–2000× *g* using a refrigerated centrifuge. After the centrifugation, plasma is immediately transferred into a clean polypropylene tube using a Pasteur pipette. The samples should be maintained at 2–8 °C while handling. Plasma is not analyzed immediately; it should be apportioned into 0.5 mL aliquots, stored at −20 °C or lower for further use [54].

#### 3.8.2. Dissection and Preparation of Brain Sample

The brain was immediately dissected out and washed with cold saline and a known amount of tissues were homogenized with an appropriate ice-cold buffer in a Teflon homogenizer. The plasma and homogenized samples were subjected to HPLC evaluation for absorbed quetiapine.

#### 3.8.3. HPLC Conditions

For the measurement of QTF using HPLC, an analytical and bioanalytical approach was devised and validated. During the in-vivo study, the proposed approach was used to estimate drug concentrations reaching the rat’s brain, plasma, and retention in the nasal mucosa. QF was evaluated using a Shimadzu LC-2010C HT high-performance liquid chromatography system (HPLC) with a UV/VIS detector and Labsolutions chromatographic software (Shimadzu, Japan). At room temperature, a reverse phase C18 column (250 × 4.6 mm, 5 l, kinetex, Phenomenex, CA, USA) was utilised. At a flow rate of 1.0 mL/min, acetonitrile and potassium dihydrogen orthophosphate buffer pH 6.0 (30:70 *v/v*) were employed as the mobile phase. The injection volume was 10 l, and the elutes were examined at a wavelength of 250 nm. In the concentration range of 1–100 g/mL, the R2 value of 0.999 was found to be linear.

#### 3.8.4. Pharmacokinetic Parameters Analysis

The WinNonLin (version 1.5, Scientific consulting, Inc., Gaithersburg, MD, USA) was used to determine pharmacokinetic parameters from the brain and plasma samples [55]. The highest plasma concentration (C_max_, µg/g) and time necessary to achieve this maximum concentration (T_max_, h) from each rat brain concentration-time curve were calculated using a non-compartmental pharmacokinetic model. According to Khallaf et al., all pharmacokinetic parameters were determined. [24]

Two indices were calculated for the brain target parameter of QTF following nasal dosing: The drug targeting efficiency percentage (% DTE) measures the relative exposure of brain to drug following I.N. administration versus systemic administration, and is computed as follows [56,57]:

(2) %DTE=(AUC0−t(brain)AUC0−t(plasma))Intranasal(AUC0−t(brain)AUC0−t(plasma))Oral×100

The % DTE value can range from − to +, with values greater than 100 indicating superior drug delivery to the brain following I.N. Nose-to-brain direct transport percentage (*DTP*%) measures the relative percentage of drug estimated to reach the brain via trigeminal or olfactory nerves (direct nose to brain route) versus overall drug delivery to the brain via the BBB and all direct routes [56,57]. The formula for calculating *DTP*% is as follows:

(3) $$DTP\%=\frac{BIN-BX}{BIN}\times 100$$

where *BIN* is the AUC_0–t_ (brain) after intranasal administration and *BX* is the brain AUC fraction provided by systemic circulation through the BBB after intranasal administration and PIN Equation (3) PIV is AUC_0–t_ of QTF in the plasma following oral administration, whereas PIN is AUC_0–t_ of QTF in the plasma following intranasal administration. The *DTP*% number ranges from − to 100%, with + values above zero indicating significant drug delivery to the brain after I.N. administration by direct nose-to-brain route, and negative values indicating efficient drug delivery to the brain via BBB permeability rather than the direct route.

### 3.9. Statistical Analysis of Results

Data were analyzed statistically using a one-way analysis of variance and represented as mean and standard deviation (ANOVA). Statistical analysis was performed using Dunnett′s t test to ascertain the values for brain uptake and pharmacokinetics. It was deemed significant at *p* < 0.05.

## 4. Conclusions

The intranasal injection of QTF-loaded lipospheres showed a shorter Tmax compared to that of intranasal and oral suspension, per the findings of an in vivo tissue distribution investigation in Wistar mice. Lipospheres were able to achieve higher drug transport efficiency (DTE%) and direct nose-to-brain drug transfer (DTP%). These reported results are consistent with our theory, according to which an intranasal route paired with liposphere technology would be a potential approach for delivering QTF to the brain directly and obtaining enough concentrations. This delivery method has a lot of potential for the treatment of schizophrenia. Our plan for the future is to compare the delivery of QTF-loaded lipospheres with other nanosystems such as transferosomes, transethosomes, and cubosomes.

## Figures and Tables

**Table 1:** The composition of different trial batches of QTF loaded lipospheres with their responses.

Formulations	Drug:Lipid	Core:Coat	%Pluronic	EE%	Vesicles Size (nm)	Zeta Potential	PDI
TB1	1:2	2:1	0.2	21.790 ± 2.0482	233.75 ± 6.8589	−24.1 ± 1.0748	0.2735 ± 0.0671
TB2	1:4	2:1	0.2	35.888 ± 1.6683	259.7 ± 7.4953	−19.45 ± 1.7111	0.542 ± 0.1555
TB3	1:6	2:1	0.2	41.670 ± 1.6187	275.9 ± 14.2835	−18.73 ± 0.5515	0.3025 ± 0.0615
TB4	1:8	2:1	0.2	65.331 ± 0.3006	294.4 ± 18.2433	−25.76 ± 0.4737	0.333 ± 0.1697
TB5	1:10	2:1	0.2	50.313 ± 1.5196	578.9 ± 34.2239	−24.05 ± 0.4101	0.824 ± 0.0721

**Table 2:** The composition of different formulations of QTF loaded lipospheres with their responses.

Formulations	Drug:Lipid	Core:Coat	%Pluronic	EE%	Vesicles Size (nm)	Zeta Potential	PDI	%Drug Released
F1	1:8	2:1	0.1	40.046 ± 1.800	436.35 ± 25.5265	−21.35 ± 2.8991	0.349 ± 0.1605	69.1164
F2	1:8	3:1	0.1	55.186 ± 0.964	489.95 ± 17.7483	−21.85 ± 0.6363	0.490 ± 0.0332	59.4658
F3	1:8	4:1	0.1	62.334 ± 0.660	528.75 ± 20.4353	−25.5 ± 1.2727	0.516 ± 0.0876	52.8374
F4	1:8	2:1	0.2	65.284 ± 0.366	294.4 ± 18.2433	−25.76 ± 0.4737	0.333 ± 0.1697	78.6064
F5	1:8	3:1	0.2	72.675 ± 0.498	321.8 ± 16.2634	−26.35 ± 1.3435	0.445 ± 0.1393	70.1822
F6	1:8	4:1	0.2	83.841 ± 1.086	338.45 ± 19.7282	−28.8 ± 1.9798	0.491 ± 0.1336	63.3056
F7	1:8	2:1	0.3	51.469 ± 1.437	546.4 ± 7.49533	−20.67 ± 1.3010	0.432 ± 0.1187	65.5978
F8	1:8	3:1	0.3	60.444 ± 1.192	563.8 ± 82.5900	−23.57 ± 2.5102	0.381 ± 0.0509	54.312
F9	1:8	4:1	0.3	71.409 ± 0.294	575.8 ± 46.6690	−26.09 ± 2.4607	0.505 ± 0.1873	43.8146

**Table 3:** Correlation Coefficient of release of QTF presented by different release kinetics equations.

Formula Code	Zero Order		First Order		Higuchi Diffusion Model		Kors–Peppas	
	R^2^	Eq	R^2^	Eq	R^2^	Eq	R^2^	Eq
F1	0.8942	y = 11.392x + 10.299	0.9599	y = −0.0871x + 1.9678	0.9677	y = 30.459x − 2.6565	0.9297	y = 78.795x + 12.312
F2	0.9242	y = 9.7096x + 7.2996	0.9697	y = −0.0651x + 1.9779	0.97217	y = 25.592x − 3.1733	0.9455	y = 66.616x + 9.2364
F3	0.8981	y = 8.7721x + 7.5736	0.9397	y = −0.0557x + 1.9715	0.96613	y = 23.382x − 2.2916	0.9331	y = 60.653x + 9.1322
F4	0.8666	y = 12.799x + 13.598	0.9585	y = −0.1142x + 1.9584	0.96266	y = 34.668x − 1.6512	0.9083	y = 88.879x + 15.715
F5	0.8909	y = 11.509x + 11.037	0.9598	y = −0.0896x + 1.9647	0.9711	y = 30.881x − 2.2225	0.9233	y = 79.472x + 13.123
F6	0.9121	y = 10.658x + 8.0304	0.9593	y = −0.0756x + 1.9766	0.9643	y = 28.165x − 3.5793	0.9455	y = 73.606x + 9.9578
F7	0.9074	y = 11.091x + 8.5314	0.9578	y = −0.0811x + 1.9756	0.9626	y = 29.36x − 3.6261	0.943	y = 76.696x + 10.499
F8	0.9197	y = 9.2261x + 6.3518	0.9568	y = −0.0593x + 1.9798	0.9626	y = 24.259x − 3.5081	0.9525	y = 63.691x + 8.0321
F9	0.9010	y = 7.5196x + 5.2225	0.9277	y = −0.0439x + 1.9804	0.9487	y = 19.831x − 2.9051	0.9467	y = 52.287x + 6.4384

**Table 4:** The effect of storage at 4°C for one month on particle size, zeta potential, and EE% of the optimum formula.

Responses	Fresh	After 7 Days	After 30 Days
Particle size (nm)	294.4 ± 18.243	296.53 ± 10.32	301.14 ± 9.27
Zeta potential (mV)	−25.76 ± 0.4737	−25.54 ± 0.98	−25.27 ± 1.04
EE%	65.284 ± 0.366	64.85 ± 1.25	63.96 ± 1.87

**Table 5:** Pharmacokinetic parameters of QTF in plasma and brain tissues after intranasal and oral administration.

Pharmacokinetic Parameter	QTF Lipospheres IN Suspension		QTF IN Suspension		QTF Oral Suspension	
	Plasma	Brain	Plasma	Brain	Plasma	Brain
C_max_ (µg/mL)	22.08 ± 10.23	237.86 ± 34.01	6.67 ± 1.37	132.37 ± 17.24	10.87 ± 0.93	190.14 ± 42.30
t_max_ (hr)	6 ± 0.25	4 ± 0.01	10 ± 0.05	4 ± 2	7.33 ± 2.31	6 ± 0.01
AUC_0-24hr_ (µg hr/g)	133.65 ± 16.5	2361.04 ± 279.46	79.09 ± 12.52	1733.93 ± 182.37	122.65 ± 9.96	1098.05 ± 39.72
AUC_0-∞_ (µg hr/g)	235.85 ± 78.53	2478.14 ± 291.32	101.64 ± 18.80	1778.05 ± 178.50	158.82 ± 23.66	1147.40 ± 68.09
MRT (hr)	29.61 ± 10.56	9.41 ± 0.47	16.99 ± 2.64	9.14 ± 0.34	16.33 ± 5.32	7.42 ± 0.50
t_1/2_ (hr)	22.07 ± 10.22	4.87 ± 1.19	8.44 ± 1.84	3.67 ± 0.54	11.32 ± 5.17	7.56 ± 2.82
DTE %		228.36		169.66		
DPT %		51.72		48.82		

## Data Availability

The data is contained in the manuscript.
